# Objectively Monitored Sleep in School‐Age Children With Cystic Fibrosis and Their Parents

**DOI:** 10.1002/ppul.71325

**Published:** 2025-10-15

**Authors:** Andrea L. Fidler, Casey Lawless, James Peugh, Dean W. Beebe, Silvia Delgado, Robin S. Everhart, David A. Fedele

**Affiliations:** ^1^ Department of Child & Adolescent Psychiatry and Behavioral Sciences Children's Hospital of Philadelphia Philadelphia Pennsylvania USA; ^2^ Division of Pulmonary & Sleep Medicine Children's Hospital of Philadelphia Philadelphia Pennsylvania USA; ^3^ Department of Psychiatry, Perelman School of Medicine University of Pennsylvania Philadelphia Pennsylvania USA; ^4^ Department of Pediatrics University of Missouri‐Kansas City Kansas City Missouri USA; ^5^ Division of Developmental and Behavioral Health Children's Mercy Kansas City Kansas City Missouri USA; ^6^ Department of Pediatrics University of Cincinnati College of Medicine Cincinnati Ohio USA; ^7^ Division of Behavioral Medicine and Clinical Psychology Cincinnati Children's Hospital Medical Center Cincinnati Ohio USA; ^8^ Pediatric Pulmonary Division University of Florida Health Gainesville Florida USA; ^9^ Department of Psychology Virginia Commonwealth University Richmond Virginia USA; ^10^ Center for Healthcare Delivery Science Nemours Children's Health Jacksonville Florida USA

## Author Contributions

**Andrea L. Fidler:** writing – original draft, data curation, investigation, conceptualization, methodology. **Casey Lawless:** investigation, conceptualization, methodology, writing – review and editing, project administration, funding acquisition. **James Peugh:** writing – review and editing, formal analysis, software. **Dean W. Beebe:** supervision, resources, writing – review and editing. **Silvia Delgado:** project administration, writing – review and editing, resources. **Robin S. Everhart:** writing – review and editing, supervision, resources, investigation. **David A. Fedele:** supervision, resources, investigation, writing – original draft, conceptualization.

## Acknowledgments

This study was supported by the Society of Pediatric Psychology Marion and Donald Routh Student Research Grant (Casey Lawless), Cystic Fibrosis Foundation Student Traineeship Grant (Casey Lawless), and an institutional training grant (T32DK063929 supporting Andrea L. Fidler).

## Conflicts of Interest

The authors declare no conflicts of interest.

To the Editor,

Children with cystic fibrosis (CF) and their parents are at risk for sleep problems, and many children with CF report a high number of sleep complaints. Compared with their peers, polysomnography studies indicate that children with CF frequently do not obtain sufficient sleep and experience several difficulties, including more waking after sleep onset (WASO) [1]. Sleep problems in children with CF are associated with decreased pulmonary function, low mood, and reduced quality of life. Additionally, although understudied in CF, sleep problems (e.g., nighttime awakenings) are common among parents of children with chronic illness and linked to poor health outcomes.

Child and parent sleep patterns are intercorrelated, leading to calls for a more holistic examination of sleep outcomes at the family level. To date, however, only a single study has examined subjective sleep problems and sleep sufficiency among dyads of children with CF and their parents [2]. This study found that most dyads reported obtaining less than the recommended levels of total sleep time (TST) and that sleep problems were positively associated within dyads [2]. The goal of the current study was to extend this study by: (1) using actigraphy to describe the sleep of children with CF and their parent, (2) examining associations between child and parent sleep, and (3) comparing objectively monitored sleep outcomes to those of non‐CF samples and national recommendations.

## 1 Methods

This study included 23 child–parent dyads. Children with CF were aged 4–12 years. Parents were required to reside with their child and be fluent in English. Children were excluded if they had a history of lung transplant or experienced an overnight hospitalization or CF exacerbation requiring oral or IV antibiotics in the past 2 weeks. We recruited dyads from CF Care Centers at the University of Florida (UF) and the Children's Hospital of Richmond at Virginia Commonwealth University (VCU). This is a secondary analysis of data from a longitudinal observational study examining sleep, disease outcomes, and quality of life in children with CF [3]. Data were collected at baseline and 6‐month follow‐up. Parents provided written informed consent, and children assented. This study was Institutional Review Board approved (UF: IRB201500549; VCU: HM20006229).

Children and parents wore ActiGraph wGTX3‐BT accelerometers on their nondominant wrists for 7 consecutive days and nights. Parents completed sleep diaries detailing both their and their child's bedtimes and risetimes each day. To be included in these analyses, dyads were required to have completed sleep diaries and worn accelerometers for ≥ 3 nights, including ≥ 1 weekend night. If dyads had data at both baseline and 6‐month follow‐up, we selected the timepoint with the most nocturnal wear‐time. Because parents did not report daytime naps, we solely focused on nocturnal sleep.

We used ActiLife (ActiGraph LLC; version 6.13.4) to score each 60 s epoch as sleep or wake using the Sadeh algorithm for children and Cole‐Kripke for adults. An inherent limitation of actigraphy research is that onset and offset can sometimes be ambiguous. We used a hierarchical approach that leveraged parent‐reported bedtime and risetimes to account for these instances. First, preliminary sleep periods were detected using the Tudor‐Locke algorithm, with a minimum sleep period of 60 min and onset/offset of 20 min. Research staff then independently examined those periods and determined final (analyzed) sleep period by applying the following rules: (1) use the preliminary algorithm‐selected sleep period if within 30 min of parent‐reported bedtime or risetime, (2) if rule 1 fails, select the closest algorithm‐determined period within 30 min of the parent‐reported time, or (3) if those fail, determine via consensus meeting (A. L. F., D. W. B.). Final sleep periods were determined by rule 1 on 37% of nights, rule 2 on 48% of nights, and rule 3 on 15% of nights.

For each night, we calculated sleep onset (time participant fell asleep), sleep offset (time participant woke), sleep period (time between sleep offset and onset), WASO (time awake during sleep period), TST (time asleep during sleep period), and sleep midpoint (time at the middle of sleep period). For each participant, we calculated social jetlag (difference between the mean sleep midpoint on weekdays vs. weekends).

We reported the mean and SD for each variable across all days and segmented by weekday versus weekend. We determined the percentage of sleep periods within the American Academy of Sleep Medicine's (AASM) recommendations: 10–13 h for 4–5 year‐olds, 9–12 h for 6–12 year‐olds, and 7+ h for adults [4]. We used dynamic structural equation modeling (DSEM) to estimate within‐dyad nightly associations between (1) child TST and parent TST and (2) child WASO and parent WASO. DSEM requires Bayesian estimation, *p* values are one‐tailed by default. We compared the sleep of our sample to that of previously published samples of children without CF [5] and parents of school‐age children [6]. The sample of children without CF was a historical control from another study, which included 46 children in the United States who were aged 6–14 years old (*M*_age_ = 10.3), classified as “medically healthy,” free from any psychiatric illness, and evenly split in terms of gender (50% female; 50% male) [5]. Our comparison parent sample consisted of 1137 Australian parents (*M*_age_ = 44.0 years; 13% male) whose sleep was assessed as part of the Longitudinal Study of Australian Children when their children were 11–12 years old [6]. Dunnett's C was used to quantify effect sizes, where 0.1 = *small*, 0.3 = *medium*, and 0.5 = *large effect*.

## 2 Results

Participants included 23 children (*M*_age_ = 7.47, range = 4–12 years; 70% male, 30% female) and 23 parents (*M*_age_ = 36.46, range = 23–59 years; 74% female, 26% male). Children had 149 nights of valid data (106 weekdays, 43 weekends); parents had 160 valid nights (115 weekdays, 45 weekends). Children averaged 6.48 nights of valid data per participant (range = 4–7) and parents averaged 5.29 nights (range = 4–10).

Children with CF averaged 07:39 ± 01:22 of TST and 86 ± 49 min of WASO (see Table 1). They failed to meet their recommended sleep periods on 57% of observed nights. Their parents had 07:31 ± 01:39 of TST, experienced 52 ± 31 min of WASO, and failed to reach the recommended ≥ 7‐h sleep period on 22% of observed nights. Both parents and children had later sleep midpoints on weekends versus weekdays (social jetlag of 46 min for children and 50 min for parents; see Supporting Information S1: Table A for average weekday vs. weekend sleep outcomes). Nightly child TST was correlated with parent TST, *r* = 0.25, *p* = 0.005. WASO was not correlated within‐dyads, *r* = 0.05, *p* = 0.33.

**Table 1:** Sleep outcomes for children with CF and their parents averaged across all days.

	Children with CF	Parents of children with CF
	*M* ± SD	*M* ± SD
Sleep onset	22:25 ± 01:38	23:09 ± 01:24
Sleep offset	07:31 ± 01:22	07:32 ± 01:48
Sleep period	09:05 ± 01:24	08:23 ± 01:46
WASO	01:26 ± 00:49	00:52 ± 00:31
TST	07:39 ± 01:22	07:31 ± 01:39
Sleep midpoint	02:58 ± 01:20	03:21 ± 01:21
Social jetlag	00:46 ± 01:15	00:50 ± 01:21

*Note:* All values are in hh:mm.

Abbreviations: social jetlag, difference between the mean sleep midpoint on weekdays versus weekend; TST, total sleep time; WASO, wake after sleep onset.

When compared with those without CF (shown in Table 2), children with CF had 42 more minutes of WASO (*C* = 0.84) and 30 less minutes of TST (*C* = 0.37). Children with and without CF had similar sleep timing (*C*s < 0.08). Parents of children with CF had similar sleep period lengths (*C* = 0.06), but had 30 min later sleep midpoints (*C* = 0.36) and 19 less minutes of WASO (*C* = 0.60) than parents of children without CF.

**Table 2:** Mean comparisons between children with and without CF.

	Children				Parents			
	Children with CF	Children without CF^a^	Difference	Dunnett's *C*	Parents of children with CF	Parents of children without CF^b^	Difference	Dunnett's *C*
Sleep onset	22:25	22:29	−00:04	0.04	23:09	22:42	+00:27	0.32
Sleep offset	07:31	07:24	+00:07	0.08	07:32	07:00	+00:32	0.30
Sleep period	09:05	08:54	+00:11	0.13	08:23	08:17	+00:06	0.06
WASO	01:26	00:44	+00:42	0.84	00:52	01:11	−00:19	0.60
TST	07:39	08:09	−00:30	0.37	07:31	07:06	+00:25	0.25
Sleep midpoint	02:58	02:57	+00:01	0.01	03:21	02:51	+00:30	0.36

*Note:* All values are in hh:mm, except for Dunnett's C, which was used to quantify effect sizes (0.1 = *small effect*, 0.3 = *medium effect*, and 0.5 = *large effect*).

Abbreviations: TST, total sleep time; WASO, wake after sleep onset.

^a^ Child healthy control group from Owens et al. [5].

^b^ Entire parent sample from Matricciani et al. [6].

## 3 Discussion

Sleep difficulties are linked to a multitude of negative outcomes, including behavior problems and decreased quality of life in children and their caregivers. Sleep disruption also increases systemic inflammation and has deleterious effects on metabolic function, which may be especially detrimental within children with CF. The current study builds upon prior work that called for sleep‐related research within the family context by being the first to use actigraphy to measure sleep outcomes among school‐age children with CF and their parents.

Consistent with previous studies that used subjective measures, we found intercorrelations in TST within parent–child dyads. Unfortunately, over half of school‐age children with CF in the current study failed to attain recommended amounts of sleep according to national guidelines [4]. While we acknowledge that inadequacy of sleep is common, school‐age children with CF were also more likely to have increased WASO and less TST compared with children without CF. Collectively, these findings corroborate previous survey‐ or polysomnography‐based studies [1] by demonstrating that school‐age children with CF are at risk for insufficient and poor‐quality sleep.

Paradoxically, we found that parents of children with CF had more TST and less WASO than parents of children without CF. Although we are unable to provide a definitive explanation, differences in sample composition may have played a role (e.g., child age, country of residence). It is possible that parents of children with CF had later bedtimes and higher sleep pressures, and subsequently more consolidated sleep, due to the demands of caring for young children with chronic medical conditions.

This study is the first to objectively measure sleep among child–parent dyads within the context of CF. A notable strength is our combined use of sleep diaries and actigraphy, given the commonly observed discrepancies between the two. However, actigraphy is only useful when worn and cannot provide details about sleep architecture. Other study limitations include the use of historic controls and a modest sample size.

We encourage CF care teams to include sleep as an area of clinical focus and periodically screen for sleep problems among patients and their families, given sleep's far‐reaching implications on psychological and physical health. Brief behavioral sleep interventions tailored for children with CF are feasible and acceptable; next steps include examining intervention efficacy. Future research should continue to explore the impact of poor sleep on health outcomes among children with CF.

## Supporting information

Supporting information.

## Data Availability

The data that support the findings of this study are available from the corresponding author upon reasonable request.

## References

[1] J. Reiter , A. Gileles‐Hillel , M. Cohen‐Cymberknoh , et al., “Sleep Disorders in Cystic Fibrosis: A Systematic Review and Meta‐Analysis,” Sleep Medicine Reviews 51 (2020): 101279, 10.1016/j.smrv.2020.101279. 32145647 PMC7198356

[2] K. C. Byars , B. Chini , E. Hente , R. Amin , and T. Boat , “Sleep Disturbance and Sleep Insufficiency in Primary Caregivers and Their Children With Cystic Fibrosis,” Journal of Cystic Fibrosis 19, no. 5 (2020): 777–782, 10.1016/j.jcf.2020.04.003. 32461045

[3] C. Lawless , “The Impact of Sleep in Children With Cystic Fibrosis” (Doctoral Dissertation, University of Florida, 2018), https://ufl-flvc.primo.exlibrisgroup.com/permalink/01FALSC_UFL/6ad6fc/alma990366928130306597.

[4] S. Paruthi , L. J. Brooks , C. D'Ambrosio , et al., “Consensus Statement of the American Academy of Sleep Medicine on the Recommended Amount of Sleep for Healthy Children: Methodology and Discussion,” Journal of Clinical Sleep Medicine 12, no. 11 (2016): 1549–1561. 27707447 10.5664/jcsm.6288 PMC5078711

[5] J. Owens , R. B. Sangal , V. K. Sutton , R. Bakken , A. J. Allen , and D. Kelsey , “Subjective and Objective Measures of Sleep in Children With Attention‐Deficit/Hyperactivity Disorder,” Sleep Medicine 10, no. 4 (2009): 446–456, 10.1016/j.sleep.2008.03.013. 18693137

[6] L. Matricciani , C. Paquet , F. Fraysse , et al., “Sleep and Cardiometabolic Risk: A Cluster Analysis of Actigraphy‐Derived Sleep Profiles in Adults and Children,” Sleep 44, no. 7 (2021): zsab014. 33515457 10.1093/sleep/zsab014

