# Supplementary material for: Objectively Monitored Sleep in School‐Age Children With Cystic Fibrosis and Their Parents
Source: Pediatr Pulmonol. 2025 Oct 15;60(10):e71325. doi: 10.1002/ppul.71325 (PMC12522017; doi:10.1002/ppul.71325)
Supplement: Supplementary file 1 — Supporting information. [file PPUL-60-0-s001.docx]

Supplementary Materials

for

Objectively Monitored Sleep in School-Age Children with Cystic Fibrosis and Their Parents

Page 1: Supplemental Table A

Page 2: Additional Relevant Citations

Supplemental Table A: Average Weekday and Weekend Sleep for Children with CF and Their Parents

|  | Children with CF on Weekdays | Children with CF on Weekends |
| --- | --- | --- |
|  | M ± SD | M ± SD |
| Sleep Onset | 22:16 ± 01:29 | 22:48 ± 01:56 |
| Sleep Offset | 07:16 ± 01:17 | 08:07 ± 01:24 |
| Sleep Period | 09:00 ± 01:25 | 09:19 ± 01:22 |
| WASO | 01:29 ± 00:48 | 01:17 ± 00:52 |
| TST | 07:30 ± 01:22 | 08:01 ± 01:18 |
| Sleep midpoint | 02:46 ± 01:11 | 03:28 ± 01:32 |
|  | Parents of Children with CF on Weekdays | Parents of Children with CF on Weekends |
|  | M ± SD | M ± SD |
| Sleep Onset | 23:03 ± 1:17 | 23:26 ± 01:38 |
| Sleep Offset | 07:11 ± 01:42 | 08:27 ± 01:46 |
| Sleep Period | 08:08 ± 01:43 | 09:01 ± 01:45 |
| WASO | 00:51 ± 00:31 | 00:54 ± 00:29 |
| TST | 07:17 ± 01:35 | 08:07 ± 01:39 |
| Sleep midpoint | 03:07 ± 01:14 | 03:56 ± 01:27 |

*Note.* All values are in hh:mm. WASO = wake after sleep onset; TST = total sleep time.

Additional Relevant Citations by Section:

Introduction

1. Naqvi SK, Sotelo C, Murry L, Simakajornboon N. Sleep architecture in children and adolescents with cystic fibrosis and the association with severity of lung disease. Sleep and Breathing. 2008;doi:10.1007/s11325-007-0123-0
2. Amin R, Bean J, Burklow K, Jeffries J. The relationship between sleep disturbance and pulmonary function in stable pediatric cystic fibrosis patients. Chest. 2005;128(3):1357-1363. doi:10.1378/chest.128.3.1357
3. Vandeleur M, Walter LM, Armstrong DS, Robinson P, Nixon GM, Horne RS. Quality of life and mood in children with cystic fibrosis: associations with sleep quality. Journal of Cystic Fibrosis. 2018;17(6):811-820.
4. Meltzer LJ, Moore M. Sleep disruptions in parents of children and adolescents with chronic illnesses: prevalence, causes, and consequences. Journal of pediatric psychology. 2008;33(3):279-291.
5. Bakırlıoğlu B, Çetinkaya B. Factors affecting sleep quality of mothers of children with chronic illnesses. Journal of pediatric nursing. 2022;66:e160-e165.
6. Mindell JA, Sadeh A, Kwon R, Goh DY. Relationship between child and maternal sleep: a developmental and cross-cultural comparison. Journal of pediatric psychology. 2015;40(7):689-696.

Methods

1. Lawless C, Fidler AL, Fritz AM, et al. The impact of sleep on physical activity in children with cystic fibrosis. Pediatric Pulmonology. 2017;52:S498-S498.
2. Sadeh A, Sharkey M, Carskadon MA. Activity-Based Sleep-Wake Identification: An Empirical Test of Methodological Issues. Sleep. 1994;17(3):201-207. doi:10.1093/sleep/17.3.201
3. Cole RJ, Kripke DF, Gruen W, Mullaney DJ, Gillin JC. Automatic sleep/wake identification from wrist activity. Sleep. 1992;15(5):461-469.
4. Panel CC, Watson NF, Badr MS, et al. Recommended amount of sleep for a healthy adult: a joint consensus statement of the American Academy of Sleep Medicine and Sleep Research Society. Journal of Clinical Sleep Medicine. 2015;11(6):591-592.

Discussion

1. Sadeh A, Gruber R, Raviv A. Sleep, Neurobehavioral Functioning, and Behavior Problems in School-Age Children. Child Development. 2002;73(2):405-417.
2. Cooper CJ, Owen PJ, Sprajcer M, et al. Interventions to improve sleep in caregivers: A systematic review and meta-analysis. Sleep Medicine Reviews. 2022;64:101658.
3. Besedovsky L, Lange T, Haack M. The Sleep-Immune Crosstalk in Health and Disease. Physiological Reviews. 2019;99(3):1325-1380. doi:10.1152/physrev.00010.2018
4. Van Cauter E, Spiegel K, Tasali E, Leproult R. Metabolic consequences of sleep and sleep loss. Sleep Med. 2008;9 Suppl 1:S23-8. doi:10.1016/S1389-9457(08)70013-3
5. Canter KS, Strang A, Wilks S, Okonak K, Chidekel A. Acceptability and feasibility of a brief behavioral sleep intervention for youth with CF. Journal of Cystic Fibrosis. 2023;22(1):179-182.
